# Whole-genome sequencing of *Chlamydia psittaci* from Australasian avian hosts: A genomics approach to a pathogen that still ruffles feathers

**DOI:** 10.1099/mgen.0.001072

**Published:** 2023-07-24

**Authors:** Vasilli Kasimov, Rhys T. White, Jonathan Foxwell, Cheryl Jenkins, Kristene Gedye, Yvonne Pannekoek, Martina Jelocnik

**Affiliations:** ^1^​ University of the Sunshine Coast, Centre for Bioinnovation, Sippy Downs, Sunshine Coast, QLD 4557, Australia; ^2^​ The University of Queensland, School of Chemistry and Molecular Biosciences, Australian Infectious Disease Research Centre, Brisbane, Queensland 4072, Australia; ^3^​ The University of Queensland, Australian Centre for Ecogenomics, Brisbane, Queensland 4072, Australia; ^4^​ Institute of Environmental Science and Research, Wellington, New Zealand; ^5^​ Animal Health Laboratory, Ministry for Primary Industries, 66 Ward Street, Upper Hutt 5018, New Zealand; ^6^​ NSW Department of Primary Industries, Elizabeth Macarthur Agricultural Institute, Menangle, New South Wales 2568, Australia; ^7^​ Massey University, School of Veterinary Science, Palmerston North 4442, New Zealand; ^8^​ University of Amsterdam, Amsterdam UMC, Department of Medical Microbiology and Infection Prevention, Amsterdam 1105, Netherlands

**Keywords:** *Chlamydia psittaci*, birds, Australia, New Zealand, culture-independent sequencing, phylogenomics, multi-locus sequence typing (MLST), novel sequence type (ST)

## Abstract

*
Chlamydia psittaci* is a globally distributed veterinary pathogen with zoonotic potential. Although *C. psittaci* infections have been reported in various hosts, isolation and culture of *Chlamydia* is challenging, hampering efforts to produce contemporary global *C. psittaci* genomes. This is particularly evident in the lack of avian *C. psittaci* genomes from Australia and New Zealand. In this study, we used culture-independent probe-based whole-genome sequencing to expand the global *C. psittaci* genome catalogue. Here, we provide new *C. psittaci* genomes from two pigeons, six psittacines, and novel hosts such as the Australian bustard (*Ardeotis australis*) and sooty shearwater (*Ardenna grisea*) from Australia and New Zealand. We also evaluated *C. psittaci* genetic diversity using multilocus sequence typing (MLST) and major outer membrane protein (*omp*A) genotyping on additional *C. psittaci *-positive samples from various captive avian hosts and field isolates from Australasia. We showed that the first *C. psittaci* genomes sequenced from New Zealand parrots and pigeons belong to the clonal sequence type (ST)24 and diverse ‘pigeon-type’ ST27 clade, respectively. Australian parrot-derived strains also clustered in the ST24 group, whereas the novel ST332 strain from the Australian bustard clustered in a genetically diverse clade of strains from a fulmar, parrot, and livestock. MLST and *omp*A genotyping revealed ST24/*omp*A genotype A in wild and captive parrots and a sooty shearwater, whilst ‘pigeon-types’ (ST27/35 and *omp*A genotypes B/E) were found in pigeons and other atypical hosts, such as captive parrots, a little blue penguin/Kororā (*Eudyptula minor*) and a zebra finch (*Taeniopygia guttata castanotis*) from Australia and New Zealand. This study provides new insights into the global phylogenomic diversity of *C. psittaci* and further demonstrates the multi-host generalist capacity of this pathogen.

## Data Summary

The study sequences are available in the National Centre for Biotechnology Information (NCBI) under BioProject accession number PRJNA820537. Raw Illumina sequence read data generated in this study have been deposited to the NCBI sequence read archive (SRA [https://www.ncbi.nlm.nih.gov/sra]) under the accession numbers SRR18532517 to SRR18532526. A complete list of SRA accession numbers is available in Table S1 (available in the online version of this article). The high-quality draft assemblies have been deposited to GenBank under the accession numbers JALPLX000000000 to JALPME000000000. The programmes used to analyse raw sequence reads for polymorphism discovery and whole-genome sequencing-based phylogenetic reconstruction are available as described in the Methods. The authors confirm that all supporting data, code, and protocols have been provided in the article or supplementary data files.

**Impact Statement**

*
Chlamydia psittaci* is a significant veterinary zoonotic pathogen with a global distribution and perhaps the broadest host range among chlamydial species. However, the challenges in isolating this intracellular pathogen have hindered efforts in producing more contemporary genomes from various hosts. To our knowledge, no publicly available *C. psittaci* genomes from New Zealand exist, despite reports of *C. psittaci* infections in New Zealand occurring since the 1940–50s. Obtaining high quality *C. psittaci* genomes from ‘real-time’ infections is possible using increasingly popular culture-independent probe-based whole-genome sequencing. Our study expands the global *C. psittaci* genomic catalogue by providing new whole-genome sequencing (WGS) data from four parrots and two pigeons from New Zealand, in addition to new WGS from two parrots and two novel avian hosts, an Australian bustard (one of Australia’s largest flying birds) and a sooty shearwater from Australia. We discovered that *C. psittaci* genomes from New Zealand parrots and pigeons cluster in the global clonal ST24 and diverse ‘pigeon-type’ ST27 clades, respectively. The novel ST332 strain from an Australian bustard clustered in a genetically diverse clade containing only four genomes from geographically distinct *C. psittaci* fulmar, parrot, and livestock strains. We also applied genotyping on additional *C. psittaci *-positive samples, revealing the generalist host nature of this pathogen, and that spillover infections from wild free-range birds to captive birds, livestock and humans are common, raising serious biosecurity concerns.

## Introduction

Over the past two decades, chlamydial genomic studies have provided essential knowledge about the phylogeny, genetic diversity, epidemiology, virulence factors, and transmission patterns of these intracellular pathogens [1–4]. However, the need for culturing these intracellular organisms has posed a significant challenge in obtaining high-quality genomes from clinical samples. The use of probe-bait capture techniques for whole-genome sequencing (WGS) of *Chlamydia* spp., including the zoonotic *Chlamydia psittaci *, has circumvented the need for culturing and allowed direct sequencing from clinical samples [5–7].

Extensive surveillance studies have revealed that *C. psittaci* has a broad host range globally (infecting >500 birds, small mammals, livestock and humans), making it the most diverse and significant veterinary pathogen with zoonotic potential of all chlamydial species [8–11]. Genomic studies further investigated and highlighted the intraspecies strain diversity of *C. psittaci *, providing novel insights into the evolution, genome content and phylogeny [1, 3, 6, 7, 12]. For example, Herrmann and colleagues successfully isolated and provided novel insights into the origin of fulmar-derived zoonotic strains from the Faroe Islands [13], whilst other genomic studies described the emergence and spillover into humans and horses of the global virulent and clonal ‘parrot-type 6BC’/sequence type (ST)24 strains [1, 6, 7, 14]. Despite the valuable insights the scarcity of such *C. psittaci* phylogenomic studies is striking, particularly when compared to the large-scale genomic analyses of *Chlamydia trachomatis* that can encompass over 500 genomes in a single study [15].

Although *C. psittaci* has historically been regarded as an avian pathogen, there are only a limited number of publicly available contemporary and historical *C. psittaci* genomes. This genomic data limitation is particularly evident for avian strains from Australia (*n*=8) and New Zealand (known as Aotearoa in the Māori language) (*n*=0). Without WGS, many global studies commonly utilise *omp*A genotyping and/or multilocus sequence typing (MLST) to provide the initial identity of infecting strains [4]. Recent studies in Australia have also detected *C. psittaci* in atypical hosts, such as the Australian white ibis (*Threskiornis moluccus*), Australian bustard (*Ardeotis australis*) and sooty shearwater (*Ardenna grisea*), with genotyping revealing that novel genetically diverse strains could be infecting atypical hosts, while ‘traditional genotypes’ are common in traditional hosts (e.g. clonal ST24*/omp*A genotype A in parrots and diverse STs*/omp*A genotypes E/B in pigeons) [6, 10, 16, 17].

Similar to Australia, avian *C. psittaci* infections have been well-recognised in New Zealand since the 1950s from diseased imported Australian parrots and their keepers [18]. Despite this, few avian surveillance studies exist on *C. psittaci* in New Zealand native and introduced birds. These studies have reported the detection of *C. psittaci* in captive and wild waterfowl [19], introduced parrots, and a range of native birds, such as the Kākā (*Nestor meridionalis*); Takahē (*Porphyrio hochstetteri*); New Zealand pigeon/Kererū (*Hemiphaga novaeseelandiae*); little blue penguin/Kororā (*Eudyptula minor*); rifleman/Tītitipounamu (*Acanthisitta chloris*); stitchbird/Hihi (*Notiomyces cincta*); whitehead/Pōpokotea (*Mohoua albicilla*), and blue duck/Whio (*Hymenolaimus malacorhynchos*) [20, 21]. However, the complete molecular characterisation of these strains is still lacking as genotyping was limited to *omp*A high-resolution melt curve analysis or to a 408 bp fragment of the *omp*A gene [19, 20]. It is worth noting that drawing inferences about genetic diversity using partial *omp*A genotyping is not necessarily congruent with MLST and genome-wide single-nucleotide variants (SNVs) [22]. To the best of our knowledge, no genomic studies nor any *C. psittaci* genomes from birds in New Zealand are currently publicly available, posing the question of the genetic diversity of the *C. psittaci* strains that are currently circulating in New Zealand and the hosts that they infect.

In this study, we applied probe-based culture-independent WGS to generate ten new *C. psittaci* draft genomes from Australian and New Zealand birds, including six parrots, two pigeons, an Australian bustard and a sooty shearwater. We aimed to expand the global collection of avian *C. psittaci* genomes and evaluate how these genomes cluster within the global *C. psittaci* phylogeny. Where WGS could not be applied, we evaluated the diversity of infecting *C. psittaci* strains from captive avian hosts and field isolates utilising MLST and *omp*A genotyping. Our findings provide new insights into the molecular epidemiology of avian *C. psittaci* strains in Australia and New Zealand.

## Methods

### Sample descriptions used for whole-genome sequencing

We employed WGS using probe-capture enrichment to provide draft genomes from ten *C *. *psittaci *-positive samples from four cultured isolates from two doves (isolated in 1985 and 2019) and two parrots (isolated in 1979 and 1984) from New Zealand, as well as *C. psittaci *-positive clinical samples from four parrots, an Australian bustard, and a sooty shearwater from Australia in 2019 and 2020 (detailed in Table S1). Notably, five Australian samples (LittleCorella, Corella_41C, Corella_42_pooled, Bustard1C and Sshearwater1_pooled) were described in our previous study [16]. For the Australian swab samples, we followed the swab processing and DNA extraction protocols previously described [6, 16]. For the isolates collected in New Zealand, DNA was extracted using standard DNA extraction protocols in the New Zealand Ministry for Primary Industries (MPI) laboratories. All samples underwent *C. psittaci *-specific qPCR to estimate the genome copy number before WGS [10, 16].

### *C. psittaci *-specific probe-based capture (Agilent SureSelect Targeted Enrichment)

WGS with probe-capture was performed utilising a set of 120-mer biotinylated RNA probes designed with the assistance of Agilent Technologies (Mulgrave, Victoria, Australia) using the Tier2 design (0.5Mbp-2.99Mbp). The probes were designed to cover the complete chromosome and plasmid of *C. psittaci *, using two reference genomes (CP3; GenBank: CP003797 and Horse_pl; GenBank: CP025423) and the plasmid pCpHorse_placenta (GenBank: CP025424), as previously described [6].

### Illumina MiSeq DNA sequencing

Following the capture of the DNA libraries, their quality was assessed using the Agilent TapeStation D1000 assay and qPCR. Subsequently, the libraries were normalised and pooled, and ten libraries were sequenced as 2×150 bp paired-end reads on the MiSeq platform using V2-300 chemistry (Illumina Inc., San Diego, California, United States) at the Australian Genome Research Facility (AGRF) (Parkville, Victoria, Australia) [6]. Nine DNA samples from six parrots, two doves, and an Australian bustard passed quality control measures, as they exhibited high loads of *C. psittaci* and had >20-fold sequence coverage and were selected for further phylogenomic analyses. WGS data for the sample Sshearwater1_pooled did not pass the quality control criteria for phylogenomic analyses. However, there was enough coverage in the read data for this sample to extract MLST and *omp*A sequences.

### Quality control of newly generated *C. psittaci* genome sequence data

The taxonomic profiling tool Kraken v2.0.7-beta [23] was used with default parameters on the paired-end reads to screen the sequencing data for contamination against the National Centre for Biotechnology Information (NCBI) Reference Sequence (RefSeq) database [24] for archaea, bacteria, human, viruses, and the ‘UniVec core’ subset of the UniVec database (a database of vector, adaptor, linker, and primer sequences). The sequence read data quality metrics of the sequence read data for all ten samples are presented in the supplementary materials (Table S2).

### Dataset curation

In addition to the ten draft genomes sequenced in this study (Table S1), 71 publicly available *C. psittaci* genomes (22 complete genomes, 49 draft genomes) were downloaded from the NCBI sequence read archive (SRA) or Assembly database as previously described [6], and were included in the subsequent analyses (Table S3). The quality metrics are outlined in Table S4.

### Quality control, *de novo* assembly, and *in silico* MLST

Before downstream analysis, the paired-end reads (FASTQ format) underwent quality control checks. Trimmomatic v0.36 [25] was used in paired-end mode to remove Illumina adaptor sequences and low-quality bases, as described previously [26]. Quality control metric reports were generated before and after read trimming using FastQC v0.11.9 (http://www.bioinformatics.babraham.ac.uk/projects/fastqc/, accessed on 25 April 2022) and collated using MultiQC v1.11 [27]. The *de novo* assemblies were achieved by inputting the paired-end reads into MGAP (https://github.com/dsarov/MGAP---Microbial-Genome-Assembler-Pipeline, accessed on 25 April 2022) and using the chromosome of the *C. psittaci* type strain 6BC (GenBank: CP002586) as a reference for scaffolding. Assembly metrics were assessed by comparing each assembly to 6BC using QUAST v5.0.2 [28]. The assembly quality metrics for all 81 genomes are summarised in the supplementary materials (Table S4). Each genome assembly’s completeness and potential contamination were assessed using CheckM v1.1.3 [29]. *In silico* MLST was done using MLST v2.19.0 (https://github.com/tseemann/mlst, accessed on 25 April 2022) with default settings to query the assemblies against the *Chlamydiales* database hosted on PubMLST [30].

### Variant detection and *C. psittaci* species level phylogenetic reconstruction

A 1 002 755 bp core-genome alignment was generated from 79 *C *. *psittaci* genomes using Parsnp v1.2 [31] and by adhering to a protocol described earlier [6] with the Horse_pl chromosome [14] serving as the reference to call SNVs. Notably, SNVs identified in highly dense SNVs and/or predicted recombination regions were removed (i.e. the plasticity zone, major outer membrane protein [*omp*A], polymorphic membrane protein [*pmp*] loci, and type III secretion system loci), similar to previous studies [3, 6, 7, 12, 14]. The filtered core-genome SNV alignment (16 595 SNVs) was run through jModelTest v2.1.10 [32], identifying the General Time Reversible (GTR) nucleotide substitution model as the best-fit evolutionary model. Maximum likelihood (ML) phylogenetic trees were reconstructed using RaxML v8.2.12 [33] (GTR-GAMMA correction) by optimising 20 distinct, randomised maximum parsimony trees before adding 1000 bootstrap replicates. We reproduced the phylogeny described above to evaluate the genetic diversity of the partial Bustard1C genome, only using 13 616 core-genome SNVs called from 78 genomes derived from a core-genome alignment of 806 180 bp. To further identify robust Phylogenetic Groups (PGs) within *C. psittaci* phylogeny, we used the 16 595 core-genome SNP alignment as input into rhierBAPS v1.0.1 [34] (an R [35] implementation of hierarchical Bayesian Analysis of Population Structure [BAPS] [36]) with one level of clustering, allowing up to ten initial clusters.

### *C. psittaci* ST24 phylogenetic reconstruction

As described above, a core-genome alignment of 986 344 bp (using 43 reference ST24 genomes and six draft genomes from this study) was generated using Parsnp, and SNVs were called against the reference chromosome Horse_pl. The alignment of 601 core-genome SNVs (including all SNVs across the chromosome) was run through jModelTest, identifying the GTR nucleotide substitution model as the best-fit evolutionary model. ML phylogenetic trees were reconstructed using RaxML (GTR-GAMMA correction) by optimising 20 distinct, randomised maximum parsimony trees before adding 1000 bootstrap replicates.

### Divergence estimates of the *C. psittaci* ST24 lineage

To calibrate the ST24 phylogeny, we used tip-dating approaches using TempEst v1.5.15 [37] and BEAST2 v2.7.1 [38]. For the Bayesian approach, we first determined whether the strict or optimised relaxed clock (with a log-normal distributed rate) model best fits our dataset. Using the tip date’s function, six models representative of a strict clock model and an optimised relaxed log-normal clock model were set up. The Bayesian skyline, coalescent constant, and exponential growth population size change models were compared for each clock model to ensure the selection of the best-fit model. The Gamma Site Model Category Count was set to four, and the GTR substitution model rates determined from jModelTest were included (i.e. rate AC=1.57, AG=3.92, AT=0.41, CG=0.62, CT=3.90, and GT=1.00). The initial clock rate was set to 1.04×10^−3^ substitutions/site/year (estimated from the root-to-tip regression analysis in TempEst) with a uniform distribution and an upper bound of 0.1. All other priors were left as default. All models were tested with the Nested Sampling Bayesian computation algorithm v1.2.0 within the BEAST2 package with a particle count of 32, sub-chain length of 5000, and Epsilon of 1.0×10^−12^. This analysis provided evidence in favour of the optimised relaxed log-normal clock model.

Once the best-fitting tree model was determined, three independent Markov chain Monte Carlo generations were conducted for 100 million generations for each analysis. Trees were sampled every 1000 generations, resulting in triplicate samples of 100 000 trees for each model test. To assess statistics, all BEAST2 runs were imported into Tracer v1.7.2 (http://github.com/beast-dev/tracer/, accessed on 21 December 2022). LogCombiner v2.7.1 (BEAST two package) then combined the replicated analyses for each model with a 10 % burn-in to assess convergence. Finally, TreeAnnotator v2.7.1 (BEAST two package) removed the 10 % burn-in and generated maximum clade credibility trees for each run (established from 243 million trees), reporting median values with a posterior probability limit set at 0.5. The resulting phylogenetic trees were visualised using FigTree v1.4.4 (http://tree.bio.ed.ac.uk/software/figtree/, accessed on 21 December 2022).

### Additional samples for MLST and *omp*A genotyping

We performed MLST and full-length *omp*A genotyping on an additional 22 new *C. psittaci *-positive samples provided by the New South Wales (NSW) Department of Primary Industries (DPI) and the New Zealand MPI as previously described [10] (Table S5). The provided samples from NSW DPI included extracted DNA from captive birds, comprising three captive (unspecified) non-poultry birds, a captive rosella (*Platycercus* spp.), three captive (unspecified) pigeons, one captive cockatiel (*Nymphicus hollandicus*), and a captive black-headed caique (*Pionites melanocephalus*). The remaining 11 samples from New Zealand included DNA extracted from 11 unknown hosts from MPI. In addition to these from our study, previously generated *omp*A and MLST data from a captive little blue penguin/Kororā, diamond dove (*Geopelia cuneata*), superb parrot (*Polytelis swainsonii*), and a zebra finch (*Taeniopygia castanotis*) was provided by Massey University (Palmerston North, New Zealand). We also used the 3 098 bp MLST, 1 085 bp full-length *omp*A gene, and 4 189 bp concatenated MLST and *omp*A gene alignments as input into rhierBAPS v1.0.1 [34] with one level of clustering (allowing up to ten initial clusters) to identify robust PGs.

### Phylogenetic and rhierBAPS analyses of *omp*A and MLST sequences

Post-amplification and Sanger sequencing of the full-length *omp*A and MLST genes, the newly generated *omp*A sequences were subjected to blast nucleotide analyses (BLASTn [39]), whilst MLST fragments and STs from additional samples were determined using the *Chlamydiales* spp. database on PubMLST [30]. The *omp*A sequences were deposited in GenBank under accession numbers: OQ709889 to OQ709910 (Supplementary materials ‘SequenceData.txt’), while STs and strains were deposited in the *Chlamydiales* spp. database on PubMLST.

The 36 concatenated STs from this study and 48 publicly available global reference *C. psittaci* STs were aligned using MAFFT v7.450 [40] (as implemented on Geneious Prime v2023.0.4 [41]), producing a 3 090 bp alignment used for ML phylogenetic tree construction (described above). Similarly, the new 36 *omp*A sequences from this study and 38 publicly available reference *omp*A sequences (which were paired with available ST data) were also aligned using MAFFT, producing a 1 085 bp alignment, which was used in ML phylogenetic tree construction (described above). Lastly, we concatenated the 3 098 bp STs to the 1085 bp *omp*A sequences for 74 strains (36 sequences from this study and 38 reference sequences with paired ST/*omp*A sequence) data to produce 4 189 bp concatenated sequences. These sequences were aligned using MAFFT, followed by a ML phylogenetic tree construction (described above).

## Results

### Genome quality assessment of newly sequenced *C. psittaci* strains

This study aimed to culture-independently sequence ten *C *. *psittaci* genomes from positive *C. psittaci* samples and isolates collected from psittacine and columbid hosts in Australia (*n*=6) and New Zealand (*n*=4), using *C. psittaci-*specific RNA probes. Of these, eight samples (six psittacine and two columbid hosts) were successfully sequenced, with the median genome size for these estimated to be 1 179 592 bp (interquartile range [IQR]: 1 175 162 to 1 180 301 bp; range: 1 171 636 to 1 183 547 bp) (Table S4). The draft assemblies exhibit a high level of contiguity, with a median N50 length of 281 184 bp (IQR: 166 475 to 328 619 bp; range: 113 369 to 383 500 bp). The quality and completeness of the eight newly sequenced draft genomes indicated a high level of completeness, with a mean genome completeness of 99.6 % and a mean contamination level of less than 1 %. A median plasmid size of 7 553 bp was resolved from nine (9/10; 90%) samples sent for WGS, whilst no chlamydial plasmid was present for sample LittleCorella. These results demonstrate the high quality of the new draft genome assemblies, characterised by high accuracy and completeness, providing a robust basis for further analysis.

The sequence data for the Bustard1C strain produced only a partial genome estimated to be 906 105 bp (composed of 11 contigs with a minimum length of 1 000 bp and an N50 value of 6 530 bp) and a complete chlamydial 7554 bp plasmid (resolved in a single contig). However, despite being a partial genome (77 % completeness), Bustard1C was utilised for phylogenetic analyses. In contrast, the sequencing of SShearwater1_pooled was suboptimal, yielding a fragmented assembly length of 706 748 bp (composed of 325 contigs with a minimum length of 1 000 bp and an N50 value of 2 447 bp) with a chlamydial 7.5 kbp plasmid resolved in two contigs. The WGS results were deemed insufficient for any further phylogenomic analyses. However, the reads from the SShearwater1_pooled sample were used to extract MLST and *omp*A sequences.

### Genomic diversity of *C. psittaci* strains from birds in Australia and New Zealand

**Fig. 1.**
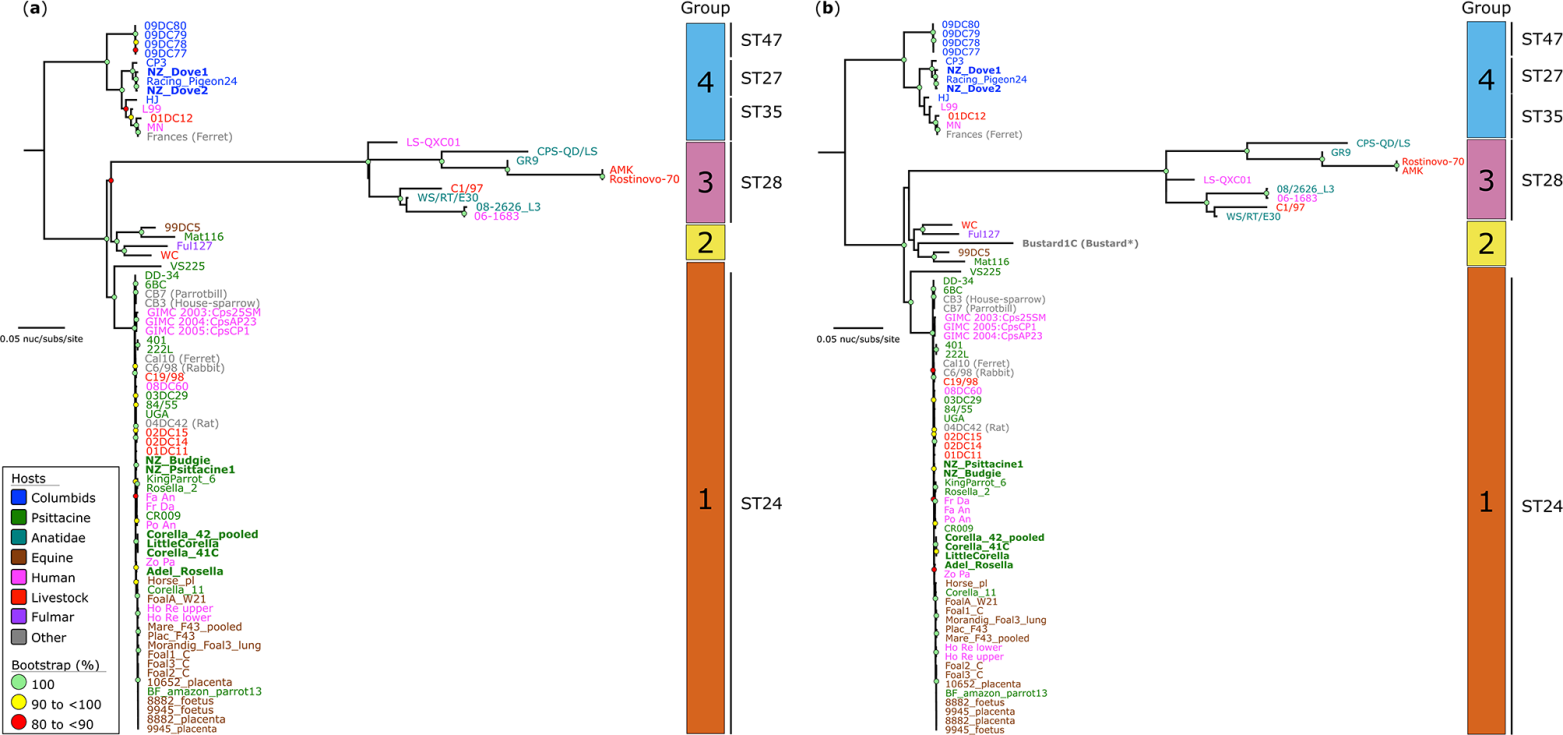
Phylogenetic relationships of *Chlamydia psittaci *. (**a**) Maximum Likelihood (ML) phylogeny inferred from 16 595 core-genome single-nucleotide variants (SNVs) called from 79 genomes. The 16 595 SNVs were derived from a core-genome alignment of 1 002 755 bp. (**b**) ML phylogeny inferred from 13 616 core-genome SNVs called from 80 genomes. The 13 616 SNVs were derived from a core-genome alignment of 806 180 bp. In both phylogenies, SNVs are called against the reference chromosome Horse_pl (GenBank: CP025423). Note, Bustard1C (*) represents a partial draft genome. Both phylogenies are rooted according to the outgroup strains M56 (GenBank: CP003795) and NJ1 (GenBank: CP003798), which have been omitted for visualisation. As indicated by the scale bar, branch lengths represent the nucleotide substitutions per site. Bootstrap values (using 1000 replicates) are shown. Strains from this study are in bold. The outer blocks reflect rhierBAPS-defined Phylogenetic Groups (PGs). Major sequence types (STs) are denoted next to the PGs.

This study evaluated the genomic diversity of eight high-quality draft *C. psittaci* genomes from four Australian psittacine hosts, two psittacine and two pigeon hosts from New Zealand, comparing them to 71 publicly available *C. psittaci* genomes (Fig. 1a). We inferred a ML phylogenetic tree from an alignment of 16 595 core-genome SNVs from 79 genomes (Fig. 1), with well-supported branches demonstrating the robustness of the evolutionary inferences made. The phylogenetic analysis showed that the 79 genomes could be grouped into four unique PGs. Of the eight genomes sequenced in the study, six (all from Australia and New Zealand psittacine-derived strains) were identified as the ST24, clustering into the globally disseminated monophyletic ST24 lineage and PG1. The New Zealand columbid pigeon strains, NZ_Dove1 and NZ_Dove2, were identified as ST27 and cluster closely with the Australian Racing_Pigeon24 and United States CP3 pigeon (ST27) strains within the broader genetically diverse ‘pigeon type’ clade and PG4.

To evaluate the genetic diversity of the strain Bustard1C, we inferred a ML phylogeny from an alignment using 13 616 core-genome SNVs from 78 genomes derived from a core-genome alignment of 806 180 bp (Fig. 1b). Similar to Fig. 1a, phylogenetic analysis shows the formation of four PGs, with Bustard1C designated as novel ST332 and clusters with other genetically and geographically distinct *C. psittaci* strains (Ful127, WC, 99DC5 and Mat116) within PG2. Within both phylogenies, the rest of the global *C. psittaci* isolates clustered into clades and PGs, as previously described [6].

### Integrating genomics and temporal information to revisit the ST24 timeline

We compared six newly sequenced high-quality draft ST24 genomes to 44 publicly available ST24 genomes. To estimate the temporal placement of the tips on the phylogenetic tree, we used the root-to-tip feature in TempEst before using the more computationally intensive BEAST2. For constructing a phylogeny of the 50 ST24 genomes, we generated a ML tree using an alignment of 653 core-genome SNVs, and then input it into TempEst (Fig. S1). GIMC 2003:Cps25SM (GenBank: CP024453) was excluded from the TempEst analysis due to the root-to-tip divergence falling outside the predicted interval for a strain sampled at that time. This discrepancy could indicate an error in the sequence data or sample metadata. Consequently, the phylogeny of the remaining 49 ST24 (excluding GIMC 2003:Cps25SM) was reconstructed using alignment of 601 core-genome SNVs (Fig. S1). The phylogeny strongly indicates the grouping of historical or archived ST24 strains associated with psittacosis (also known as ornithosis or parrot fever) outbreaks in the 1930s to 1950s, and the contemporary or emerging ST24 strains, as a monophyletic clade. The analysis estimated that the divergence between these two groups occurred between 141 and 91 years ago.

**Fig. 2.**
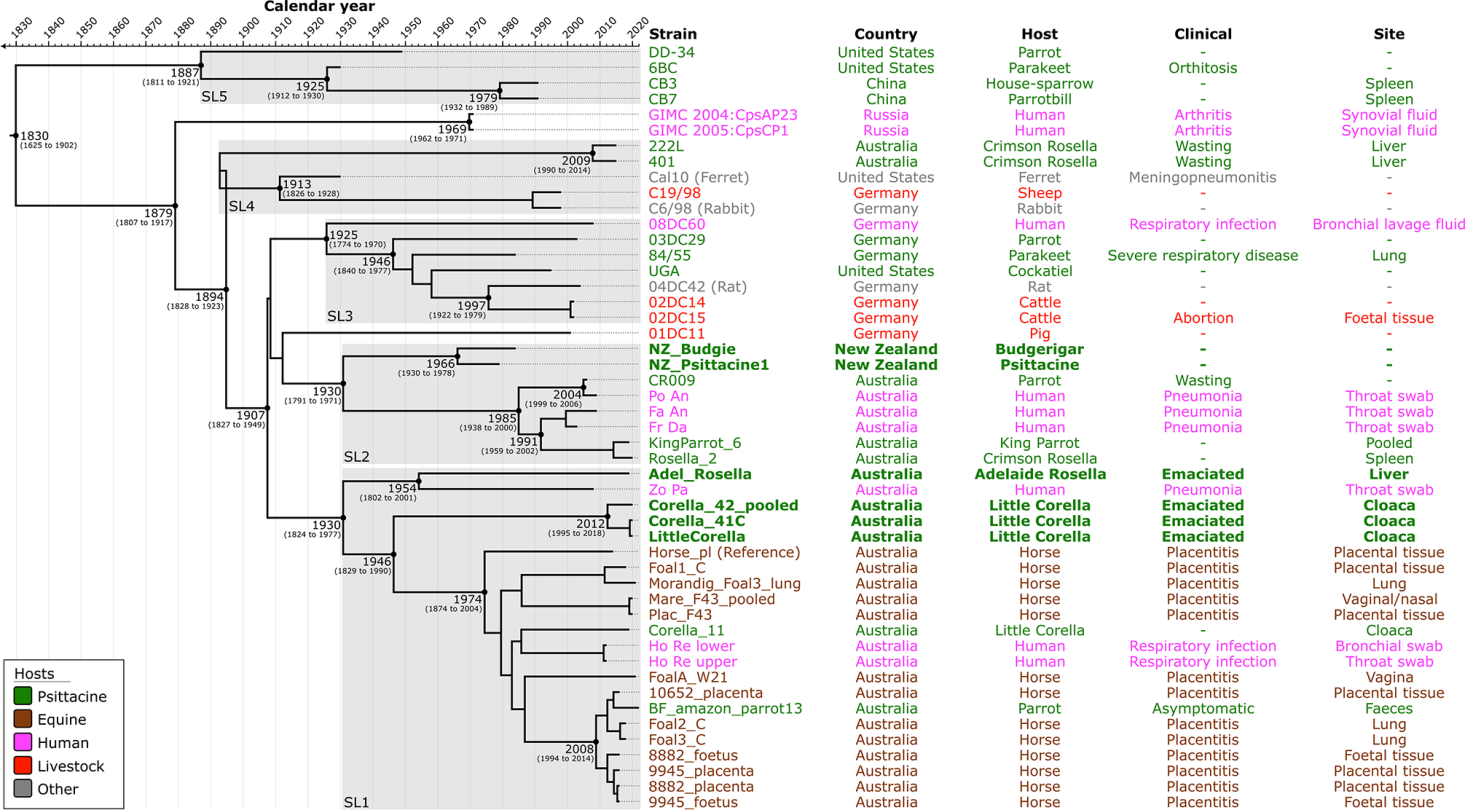
Evolutionary reconstruction of *Chlamydia psittaci* sequence type (ST)24. A time-calibrated maximum clade credibility tree was inferred from 601 core-genome single-nucleotide variants (SNVs) from 49 ST24 genomes. SNVs were derived from a core-genome alignment of 986 344 bp and are called against the reference chromosome Horse_pl (GenBank: CP025423). The X-axis represents the emergence time estimates. Strains from this study are in bold. Major ST24 sublineages (SL)1–5 are shown.

After detecting an appropriate temporal signal in the ST24 dataset (*n*=49), we used the Nested Sampling Bayesian computation algorithm to infer the best-fitting tree model and produce a time-calibrated phylogeny. The results of the Nested Sampling algorithm favoured the relaxed log-normal clock model combined with the Bayesian skyline population size change model, with a marginal likelihood estimate of −4538.79 (standard deviation: ±2.69) (Table S6). The BEAST2 analysis further confirmed for the evolutionary relationships inferred from this *C. psittaci* ST24 dataset (Fig. 2). Most internal nodes in the maximum clade credibility tree have posterior probabilities greater than 0.95, indicating high confidence in the inferred evolutionary relationships (Fig. S2). Additionally, the effective sample size for all parameters in the model is greater than 250, indicating successful convergence of the Markov chain Monte Carlo chains and robust estimates. The BEAST2 analysis, using median node heights, estimates the time of the most recent common ancestor (MRCA) of the ST24 lineage as 1830, with a 95 % highest posterior density (HPD) interval of 1625 to 1902 (Fig. 2). The median evolutionary rate is 5.58×10^−4^ substitutions per site per year with a 95 % HPD range of 1.19×10^−4^ to 9.10×10^−4^. This ST24 dataset describes one SNV for every 1640.9 bases across the 986 158 bp core-genome to correct for ascertainment bias. This results in a genome-wide mutation rate of 3.45×10^−7^ mutations/year/site, relative to genome size, for the ST24 lineage, in concordance with previous studies [1, 6, 7].

### Evolutionary timeline of avian ST24 sublineages from Australia and New Zealand

BEAST2-based phylogenetic inference resolved fine-detail relationships among ST24 strains, establishing five ST24 major sublineages (SL1 to SL5), as previously described [6]. The MRCA of all five major SLs emerged circa 1830 (95 % HPD: 1625 to 1902) (Fig. 2). Interestingly, Australian parrot-derived strains from this study clustered within the SL1 and SL2, while the New Zealand parrot-derived strains clustered exclusively in SL2. SL1, containing 22 Australian genomes, remained the predominant ST24 sublineage (emerged circa 1930 [95 % HPD: 1824 to 1977]).

In SL1, four avian-derived strains from this study formed two distinct clusters. Strain Adel_Rosella, identified in 2019 from an Adelaide rosella (*Platycercus elegans adelaidae*) in South Australia, formed a distinct cluster (emerged circa 1954 [95 % HPD: 1802 to 2001]) with the human-derived strain Zo Pa, isolated in 2008 in NSW, Australia. The remaining three avian strains within SL1 were collected from wild little corellas (*Cacatua sanguinea*) from a distinct region in Queensland (QLD), Australia, in 2020, forming their own cluster (emerged circa 2012 [95 % HPD: 1995 to 2018]). Notably, the genome BF_amazon_parrot13, from a captive blue-fronted amazon parrot (*Amazona aestiva*) from Victoria (VIC), Australia, clustered with seven horse strains from NSW, in a subclade estimated to have emerged circa 2008 (95 % HPD: 1994 to 2014). The two *C. psittaci* strains isolated from New Zealand parrots (NZ_Budgie and NZ_Psittacine1) were placed in a distinct subcluster within SL2, emerging around 1930 (95 % HPD: 1791 to 1971). SL2 also included *C. psittaci* genomes from three humans and three parrots collected from the *C. psittaci* endemic Blue Mountains region in NSW, Australia.

SL3, SL4 and SL5 are more genetically diverse, and geographically distinct sets of *C. psittaci* strains, none of which cluster with any strains collected from this study. SL3 (emerged circa 1925 [95 % HPD: 1774 to 1970]) encompasses seven genomes from a rat (04DC42), two cattle (02DC14 and 02DC15), one human (08DC60), and three parrots (03DC29, 84/55 and UGA) collected between 1984 and 2008 from Germany and the United States. SL4 emerged circa 1893 and contains two distinct clusters, one comprising two crimson rosellas strains (222L and 401) from VIC, Australia, collected in 2015, whilst the second comprising genomes collected between 1930 and 1998 from a German sheep (C19/98) and rabbit (C6/98), and a ferret (Cal10) in the United States. Caution is advised when interpreting the MRCA of SL4. As the maximum clade credibility tree was generated with median node heights, it shows a ‘negative branch length’, suggesting the descendant node of SL4 is older than its direct ancestor (Fig. 2). This is likely due to the low frequency of the clade, which causes the median heights of adjacent nodes to be derived from different sets of trees without a direct ancestor-descendant relationship. Lastly, SL5 (emerged circa 1887 [95 % HPD: 1811 to 1921]) contains two parrot strains from the United States, including the first genome 6BC (collected in 1930) and DD-34 (collected in 1949), in addition to two passerine strains from China, collected in 1991 (CB3 from a parrotbill and CB7 from a house-sparrow).

### Exploring avian *C. psittaci* strain diversity using MLST and *omp*A genotyping

**Fig. 3.**
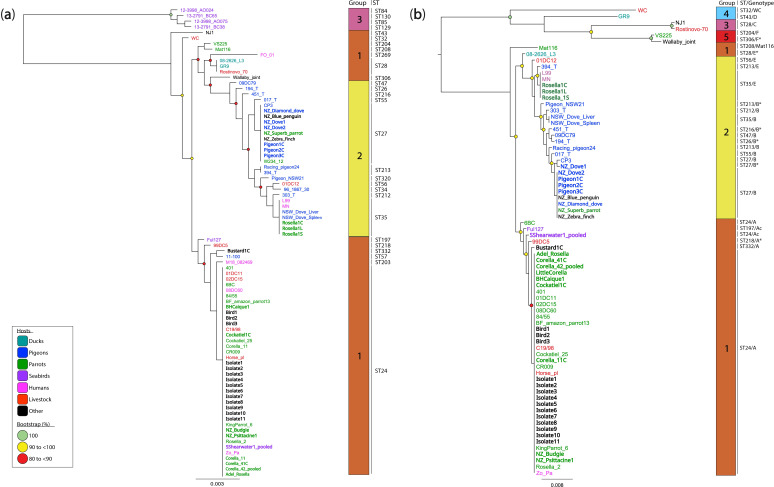
Maximum likelihood (ML) phylogenetic analysis of (**a**) 3 098 bp alignment representing concatenated multilocus sequence typing (MLST) sequences, including 36 from this study and 48 reference MLSTs; and (**b**) 4 189 bp alignment of the concatenated paired MLST and *omp*A sequences, including 36 from this study and 38 reference sequences. Both phylogenies are midpoint rooted. Branch lengths represent the nucleotide substitutions per site, as indicated by the scale bar. Bootstrap values (using 1000 replicates) are shown. Strains from this study are in bold. Sequences are coloured according to their respective hosts. The outer blocks reflect rhierBAPS-defined Phylogenetic Groups (PGs). Major ST is denoted next to the PGs.

In the absence of WGS, MLST supplemented with *omp*A genotyping was applied to 26 additional *C. psittaci* sequences from captive birds and field isolates from Australia and New Zealand to assess the genetic diversity of the newly typed clinical strains. Using MLST-derived phylogeny, rhierBAPS resolved three PGs [1–3], where (i) PG1 included the clonal ST24 and other genetically distinct STs; (ii) PG2 included genetically diverse ‘pigeon’ STs, and (iii) PG3 included genetically diverse STs detected in Northern gannets (*Morus bassanus*) (Fig. 3a).

In our sample set, clonal ST24/*omp*A genotype A was the most frequently detected genotype, identified in 22/36 (61.11 %) samples, including those from a captive cockatiel and black-headed caique, wild parrots from Australia and 13 psittacine and field isolates from New Zealand. *C. psittaci* ST24 was detected from a sooty shearwater but with *omp*A genotype Ac (*omp*A gene 100 % identical to strain Ful127) (Fig. 3a; Table S1). Surprisingly, clonal ST27/*omp*A genotype B/B* was detected in 9/36 (25.0 %) samples from diverse avian hosts, including those from three captive pigeons from Australia, and two doves, a captive diamond dove, a zebra finch, a superb parrot, and a little blue penguin/Kororā from New Zealand. In this study, for the first time, the ‘pigeon-type’ ST35/*omp*A genotype E was detected in 3/36 (8.33 %) samples from a captive rosella from Australia. These ‘pigeon-type’ strains (ST27 and 35) clustered with other closely related strains from pigeons, humans, and a sulphur-crested cockatoo from Australia. Lastly, *C. psittaci* detected in samples from an Australian bustard was resolved as a novel ST332 with *omp*A genotype A, clustering with a pigeon ST57 strain (Fig. 3a; Table S5). The remaining reference STs from other diverse hosts clustered within their own lineages (Fig. 3a).

### Phylogenetic analyses of concatenated *omp*A and MLST sequences

Although *omp*A genotyping shows some associations with host tropism, it is not necessarily congruent with whole genome-based phylogeny, unlike MLST. By comparing phylogenetic trees constructed from MLST and *omp*A sequences from samples in this study, discrepancies in phylogenetic clustering were observed (Fig. S3). Therefore, to assess whether MLST supplemented with *omp*A genotyping could provide further phylogenetic resolution without WGS data, we constructed a ML tree from a 4 189 bp alignment of concatenated paired MLST and *omp*A sequences from 36 sequences from this study and 38 reference sequences (Fig. 3b).

All ST24/*omp*A genotype A strains formed a well-supported monophyletic sub-clade, clustering in a larger clade with closely related diverse STs and *omp*A genotype A variants, including strains 6BC, Ful127, SShearwater_pooled and 99DC5. These, in addition to ST28/*omp*A genotype E and ST208/*omp*A genotype Mat116, all formed PG1. PG2 clustered all the ‘pigeon-type’ STs/*omp*A genotypes E, B, and E/B variants, where ST27/*omp*A genotype B and ST35/*omp*A genotype E formed monophyletic subclades. PG3 included ST28/*omp*A genotype C, whilst PG4 and PG5 included other diverse STs (32, 43, 204 and 306) and *omp*A genotypes (D, F, F* and WC) (Fig. 3b).

## Discussion

This study presents eight near-complete (up to 99 % completeness) and one partial (77.6 % completeness) draft *C. psittaci* genomes obtained from diverse avian species in Australia, and particularly New Zealand, where *C. psittaci* WGS data has not been previously available. Finally, in the absence of WGS, we also applied MLST and *omp*A genotyping on clinical samples from diverse captive avian hosts and field isolates, discovering traditional genotypes in non-traditional hosts. We again highlight the vast host range and ongoing diversity of *C. psittaci* globally.

### WGS of Australasian *C. psittaci* strains from avian hosts further highlights both strain diversity and clonality

Although *C. psittaci* is somewhat inconsistently referred to as an avian pathogen, a severe dearth of publicly available genomes isolated from avian hosts exist, demonstrated by only 28/71 (39.4 %) of reference genomes being from avian hosts, with most strains described before 2015. Secondly, there is a notable lack of genomic data from genetically diverse avian lineages, as 16/28 (57.1 %) genomes from avian hosts used in this study belong to the ST24 lineage. This is further exemplified by the lack of genomes from ‘pigeon-type’ strains (with only seven publicly available genomes) and other clinically relevant avian strains, such as the single genome from ST197 strain Ful127 detected in fulmars from the Faroe Islands, which previously caused an epidemic of psittacosis/ornithosis and resulted in a 20 % mortality rate [13, 42]. To combat these challenges, extensive sampling followed by WGS of *C. psittaci *-positive samples is needed from a broader range of atypical hosts, as shown by the approach utilised in our previous sampling [16] and genomic [6] studies.

We also expanded the catalogue of underrepresented and diverse *C. psittaci* genomes. Briefly, the genomes of two New Zealand ST27 pigeon strains were diverse yet closely related to the other pigeon ST27 strains, indicating some degree of clonality within this subclade, similar to that observed for the pigeon ST47 subclade. Furthermore, host tropism within the ‘pigeon-type’ PG4 seems more evident [12], with human and historical ferret-derived strains likely resulting from infection spillover from pigeons. However, these observations can only be adequately assessed when additional genomes from ‘pigeon-type’ subclades are provided. Another interesting finding is that the partial core-genome of novel ST332, detected in an Australian bustard, formed its own lineage within a genetically diverse clade consisting of only four genomes from a fulmar (Ful127), horse (99DC5), cattle (WC) and parrot (Mat116). Nevertheless, despite obtaining a partial genome, the regions in the draft genome of Bustard1C sequenced reliably contained phylogenetically informative SNVs, enabling a robust placement within a global context.

While our focus was mainly on the phylogenomic diversity and relationships between avian and other host strains, a recent comparative genomic study by Sachse and colleagues thoroughly investigated *C. psittaci* genomic variable areas associated with phylogeny. Particularly for atypical strains (such as Ful127, Rostinovo-70, AMK), the areas driving phylogeny can include *pmp* (polymorphic membrane protein), *Inc* (inclusion membrane protein) and the PZ (plasticity zone) genes, in addition to unique sequences acquired due to recombination [12]. Cell biology studies also showed that different infectious potential and host tropism of avian and mammalian *C. psittaci* strains is associated with differences in their *pmp* gene repertoires [43]. Nevertheless, coupling surveillance studies with WGS (wherever possible) will assist future epidemiological studies, and help capture spillover events of *C. psittaci* strains.

### Revised ST24 evolutionary timelines suggest Australian parrots may have caused horse infections in Australia and potentially introduced *C. psittaci* to New Zealand

Using robust molecular clock analysis of 49 ST24 genomes, we observed that the genomes of the two psittacine ST24 strains from New Zealand clustered in a distinct subclade (emerged circa 1966) together with other Australian psittacine and human strains in SL2. The MRCA for SL2 was circa the 1930s, similar to that of SL1 (strictly containing Australian-only human, parrots and horse-derived strains), also emerging circa 1930s. Australia has long been known as the ‘Land of Parrots’ [44], with parrot *C. psittaci* infections characterised since the early 1930s by Sir Frank Macfarlane Burnet and colleagues [45, 46]. It has been reported that *C. psittaci* was first isolated in New Zealand in 1953 from several imported and presumably infected Australian parrots that were also causally linked to several human cases of psittacosis/ornithosis [18, 47]. Since then, *C. psittaci* has been regularly reported in avian infections in New Zealand [19–21]. Although this supports the timeline from our study, any phylogenomic dating should be interpreted carefully, particularly with a lack of additional *C. psittaci* ST24 genomic data from New Zealand.

In Australia, *C. psittaci* has been well-reported as a cause of reproductive loss in mares, with the hypothesis that *C. psittaci* spilled over from parrots to horses [6, 10]. However, we can perhaps provide further (more confident) evidence to support this hypothesis. Previously, we showed that all Australian equine *C. psittaci* strains clustered into SL1 (emerged circa 1979) with other Australian psittacine and human strains, but also forming subclades consisting of equine strains only [6]. In this study, additional parrot ST24 genomes (from a wild rosella, three corellas, and a captive parrot) clustered in the broader SL1 (similarly emerging circa 1974). More specifically, a recently sequenced *C. psittaci* from a captive blue-fronted amazon parrot (BF_amazon_parrot13) clustered within the ‘horse-only’ subclade (emerged circa 2008) (Fig. 2).

In Australia, many native avian species, including the little corella (a common host for *C. psittaci* [10, 16, 48]), have become problematic agricultural pests [49]. Open pastures with unrestricted access to grain and water can attract flocks of little corellas to farmlands [50], potentially facilitating the transmission of *C. psittaci* to various Australian livestock species, including horses. However, *C. psittaci* strains from little corellas clustered in distinct subclades (with no horses) of the clonal ST24 SL1 (emerged circa 1974 and 2012) (Fig. 2). Similarly, our current and previous study suggests that the captive parrot may have been infected with strains from other wild-caught parrots introduced into the local avian pet trade [51]. To extrapolate the origin and spillover direction of infections in captive and wild parrots, livestock and humans, more *C. psittaci* genomic data is still needed.

### *C. psittaci* genotyping: a useful molecular epidemiology tool that is here to stay

In contrast to the limited number of comprehensive phylogenomic and comparative genomic studies exploring diverse *C. psittaci* lineages, many global genotyping and surveillance studies have demonstrated substantial strain and host diversity [10, 19, 52–56]. Routinely employing culture-independent WGS in diagnostic laboratories and research is still costly (e.g. *C. psittaci* Agilent probes and WGS costs in this study were estimated at AUD$17 000 for 16 samples). Comparatively MLST (combined with *omp*A genotyping) is a cost-effective and less technical tool for chlamydial molecular epidemiology studies [4, 6, 10]. This study additionally obtained 26 *C *. *psittaci *-positive samples from captive and wild birds from Australia and New Zealand for MLST/*omp*A genotyping (Fig. 3).

Unsurprisingly, most of the genotyped samples were identified as clonal ST24/*omp*A genotype A, including all New Zealand field isolates, captive non-poultry birds, a captive cockatiel and a captive black-headed caique from Australia. These results further highlight the vast dispersal of the clonal ST24 lineage within captive and wild avian hosts. We further identified the ‘pigeon-type’ *C. psittaci* strains (ST35/*omp*A genotype E) in samples collected from a captive rosella, similar to a strain previously reported in an Australian spotted dove (*Spilopelia chinensis*) [10]. Sequences from Australian captive pigeons and a captive little blue penguin/Kororā, superb parrot, diamond dove, and zebra finch from New Zealand were identified as the ‘pigeon-type’ ST27/*omp*A genotype B, previously identified in feral Swiss pigeons [52, 55], but also a wild sulphur-crested cockatoo and a horse from Australia [6]. Our study provides further evidence of the potential host-switching and spillover potential of ‘pigeon-type’ strains to parrots and other unconventional (non-psittacine) hosts.

Whilst WGS was unsuccessful for the strain SShearwater1_pooled (detected in a sooty shearwater from Australia), MLST and *omp*A genotyping resolved this strain as ST24/*omp*A genotype Ac, sharing an identical *omp*A genotype to strain Ful127 identified in birds (fulmars) of the same taxonomic order (Procellariformes). Shearwaters are one of Australia’s most abundant seabirds, known to undertake long-distance migrations (over 74 000 kilometres) to and from New Zealand and other regions, including Alaska, California, and Japan, potentially contributing to the importation and exportation of *C. psittaci* and other zoonotic pathogens to and from our local environment [16, 57].

MLST supplemented with *omp*A genotyping revealed atypical variants, as observed in strains SShearwater1_pooled (ST24/*omp*A genotype Ac) and Bustard1C (novel ST332/*omp*A genotype A). We further sought to resolve phylogenetic relationships between strains by aligning concatenated MLST sequences with full-length *omp*A sequences. In these analyses, we more clearly observed that strains SShearwater1_pooled (ST24/*omp*A genotype Ac) and Bustard1C (novel ST332/*omp*A genotype A) formed their own distinct lineages and are related to ST197/*omp*A genotype Ac (detected in fulmars) and clonal ST24/genotype A strains. The emergence of chlamydial variants with virulence-associated genome backbone but a prevalent *omp*A genotype (as observed in *C. trachomatis* atypical genotypes) may affect transmission, tissue tropism and pathogenic potential [58]. Therefore, concatenating *C. psittaci* MLST and *omp*A sequences (in the absence of WGS) may provide even finer evolutionary detail.

### What about *C. psittaci* strains from South America?

The high desirability of parrots as pets, attributable to their intelligence, plumage, and engaging social behaviours, led to their extensive export and global trade during the 1930s, ultimately sparking the widespread outbreak of what is now recognised as the ‘Great Parrot Fever’. This *C. psittaci* pandemic affected several countries, including Argentina, the United Kingdom, the United States, and Germany, with hypothesised origins in South America and/or Australia [1, 7, 59, 60]. However, few *C. psittaci *-specific surveillance and genotyping studies (and no genomes sequenced from the South Americas) exist to date [8]. Among the limited number of South American studies conducted, the majority only performed partial *C. psittaci omp*A characterisation [61–65], of which *omp*A genotype A was detected the most, followed by a lower frequency of genotypes B, E/B and WC. To contextualise these findings within a global phylogenetic framework and to estimate strain emergence, additional WGS of *C. psittaci* strains from South America is required, particularly from the clonal ST24 lineages and genetically distinct E/B and WC genotypes (in which little WGS data currently exists).

## Conclusion

In conclusion, this study provides an additional nine quality draft genomes from parrots and pigeons, and a novel avian host (Australian bustard), whilst additionally providing the first genomic insights into *C. psittaci* strains from New Zealand. Furthermore, we provide additional evidence supporting the spillover of the clonal sequence type (ST)24 parrot strains to Australian horses. Genotyping of clinical samples revealed further interesting observations about the genetic diversity and host range of *C. psittaci* in Australia and New Zealand. This included the ST24/*omp*A genotype A variant characterised in a sooty shearwater and ‘pigeon-type’ ST35 and ST27 observed in non-pigeon hosts (captive parrots, a little blue penguin/Kororā and zebra finch) demonstrating the strong host-switching abilities of *C. psittaci *. Obtaining more WGS data from atypical hosts such as migratory water birds, small mammals, and other livestock species would help further capture the global diversity of *C. psittaci *, and estimate the emergence dates of more genetically diverse strains.

## Supplementary Data

Supplementary material 1

Supplementary material 2
