# Supplementary material for: Whole-genome sequencing of Chlamydia psittaci from Australasian avian hosts: A genomics approach to a pathogen that still ruffles feathers
Source: Microb Genom. 2023 Jul 24;9(7):mgen001072. doi: 10.1099/mgen.0.001072 (PMC10438822; doi:10.1099/mgen.0.001072)
Supplement: Supplementary material 1 [file mgen-9-1072-s001.pdf]

SUPPLEMENTARY APPENDIX

Whole-genome sequencing of *Chlamydia psittaci* from Australasian avian hosts: A genomics approach to a pathogen that still ruffles feathers

Author names

Vasilli Kasimov[1#], Rhys T. White[1-4#], Jonathan Foxwell[5], Cheryl Jenkins[6], Kristene Gedye[7], Yvonne Pannekoek[8], Martina Jelocnik[1]

Affiliation

[1]University of the Sunshine Coast, Centre for Bioinnovation, Sippy Downs, Sunshine Coast, QLD 4557, Australia

[2]The University of Queensland, School of Chemistry and Molecular Biosciences, Australian Infectious Disease Research Centre, Brisbane, Queensland 4072, Australia

[3]The University of Queensland, Australian Centre for Ecogenomics, Brisbane, Queensland 4072, Australia

[4]Institute of Environmental Science and Research, Wellington, New Zealand

[5]Animal Health Laboratory, Ministry for Primary Industries, 66 Ward Street, Upper Hutt 5018, New Zealand

[6]NSW Department of Primary Industries, Elizabeth Macarthur Agricultural Institute, Menangle, New South Wales 2568, Australia

[7]Massey University, School of Veterinary Science, Palmerston North 4442, New Zealand

[8]University of Amsterdam, Amsterdam UMC, Department of Medical Microbiology and Infection Prevention, Amsterdam 1105, The Netherlands

#: These authors have contributed equally.

Corresponding author

*Corresponding author: Martina Jelocnik, University of the Sunshine Coast, Centre for Bioinnovation, Sippy Downs, Queensland 4557, Australia;

Telephone: +61-7-5456-3585; Email: mjelocni@usc.edu.au

Keywords

Chlamydia psittaci; birds; Australia; New Zealand; culture-independent sequencing; phylogenomics; multi-locus sequence typing (MLST); novel sequence type (ST)

<u>This file includes:</u>

Supplementary Figure S1. Maximum likelihood evolutionary reconstruction of *Chlamydia psittaci* sequence type (ST)24.

Supplementary Figure S2. Evolutionary reconstruction of *Chlamydia psittaci* sequence type (ST)24.

Supplementary Figure S3. Tanglegram comparing MLST and full-length *omp*A gene sequences.

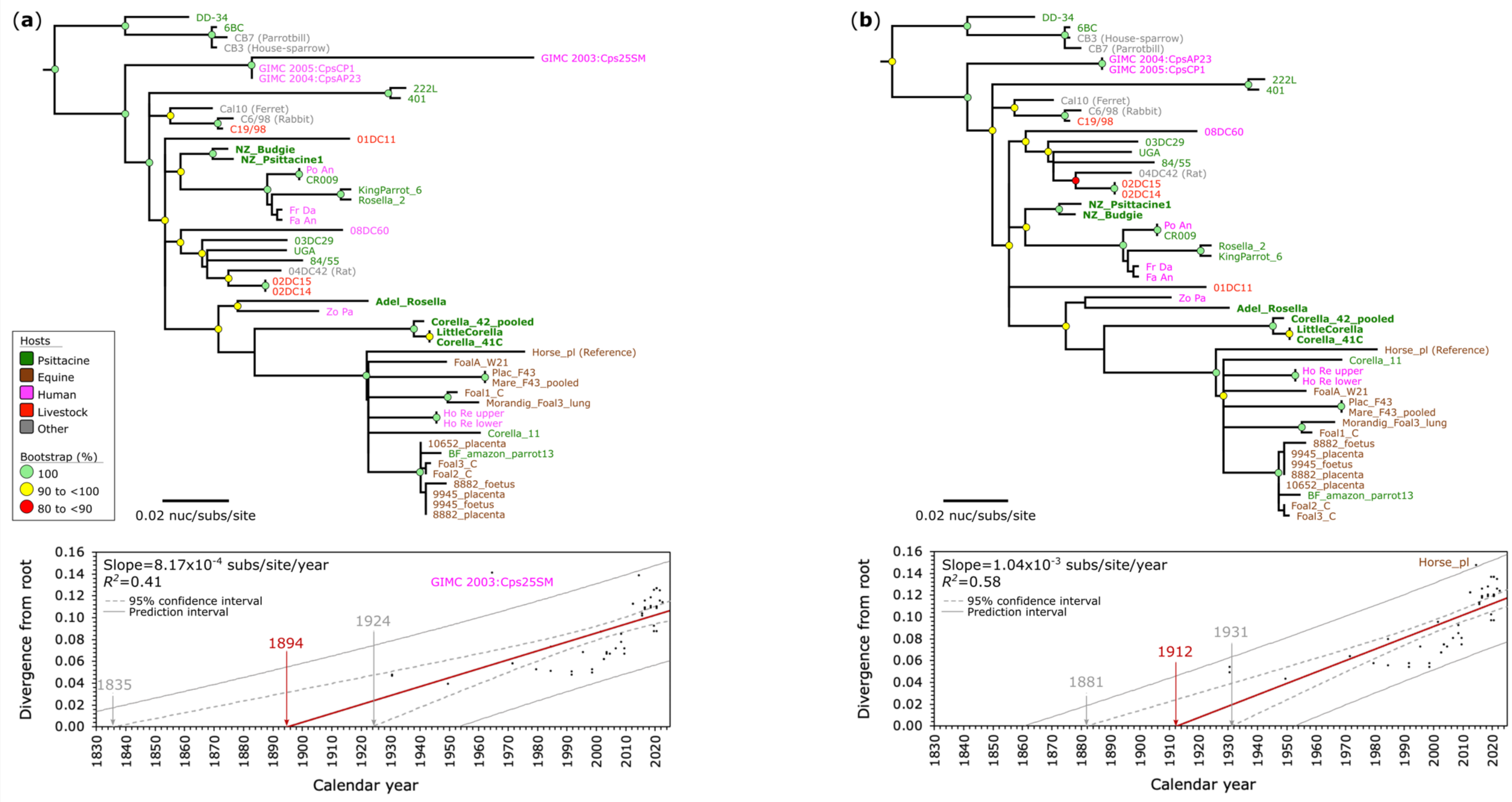

Supplementary Figure S1. Maximum likelihood evolutionary reconstruction of *Chlamydia psittaci* sequence type (ST)24. (a) Phylogeny was inferred from 653 core-genome single-nucleotide variants (SNVs) called from 50 genomes. The 653 SNVs were derived from a core-genome alignment of 993,002 bp. (b) Phylogeny was inferred from 601 core-genome SNVs called from 49 genomes. The 601 SNVs were derived from a core-genome alignment of 986,344 bp. In both phylogenies, SNVs are called against the reference chromosome Horse_pl (GenBank: CP025423). Both phylogenies are rooted according to the outgroup strains VS225 (GenBank: CP003793), which has been omitted for visualisation. Branch lengths represent the nucleotide substitutions per site, as indicated by the scale bar. Bootstrap values (using 1000 replicates) are shown. Strains from this study are in bold. Below each phylogeny is a linear regression of root-to-tip genetic distance plotted against the collection year implemented in TempEST. The slope of the solid red regression line indicates the nucleotide substitution rate.

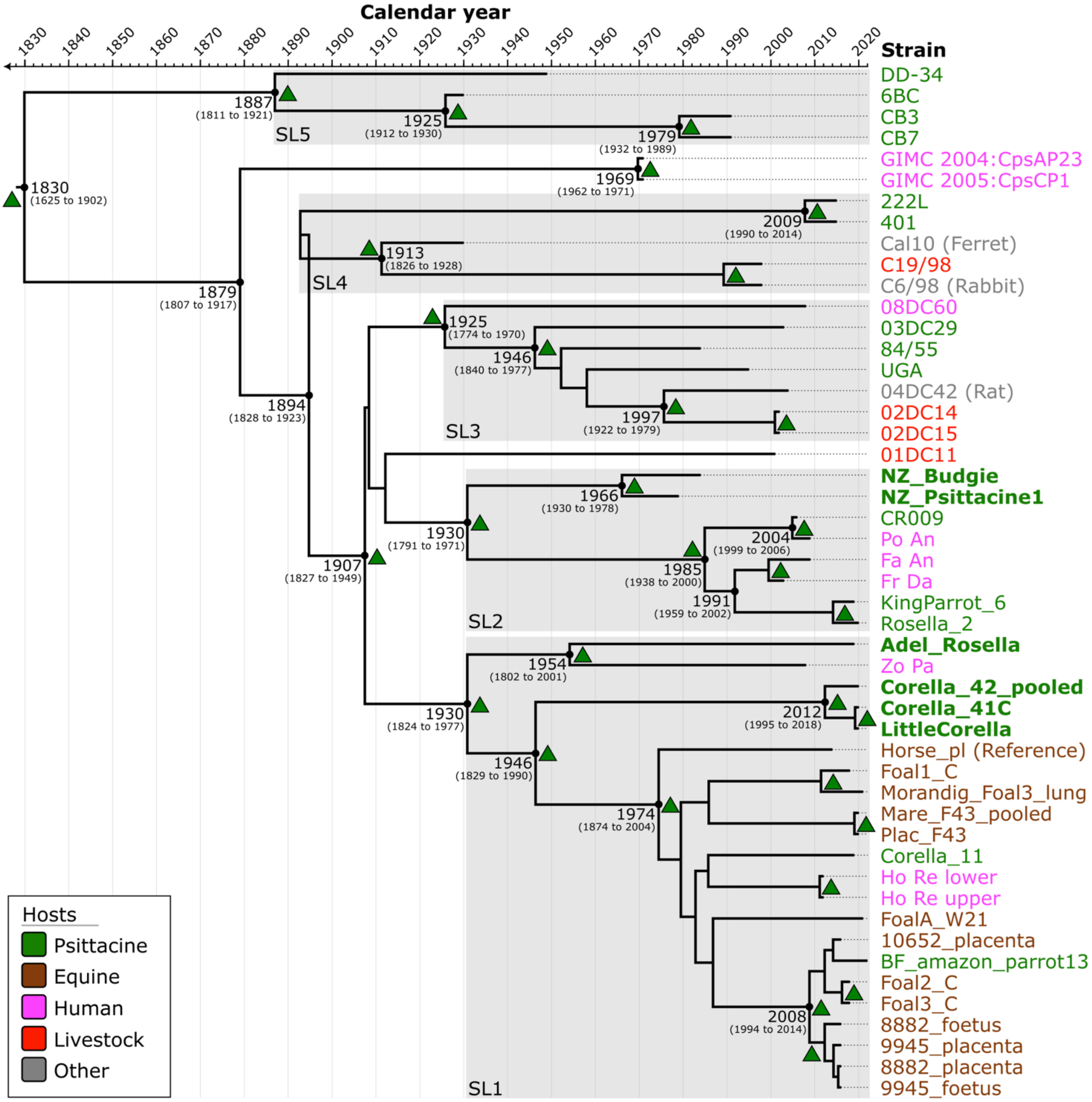

Supplementary Figure S2. Evolutionary reconstruction of *Chlamydia psittaci* sequence type (ST)24. A time-calibrated maximum clade credibility tree was inferred from 601 core-genome single-nucleotide variants (SNVs) called from 49 ST24 genomes. SNVs were derived from a core-genome alignment of 986,344 bp and are called against the reference chromosome Horse_pl (GenBank: CP025423). The X-axis represents the emergence time estimates. Strains from this study are in bold. Major ST24 sub-lineages (SL)1–4 are shown. The green triangles represent nodes with posterior probabilities greater than 0.95.

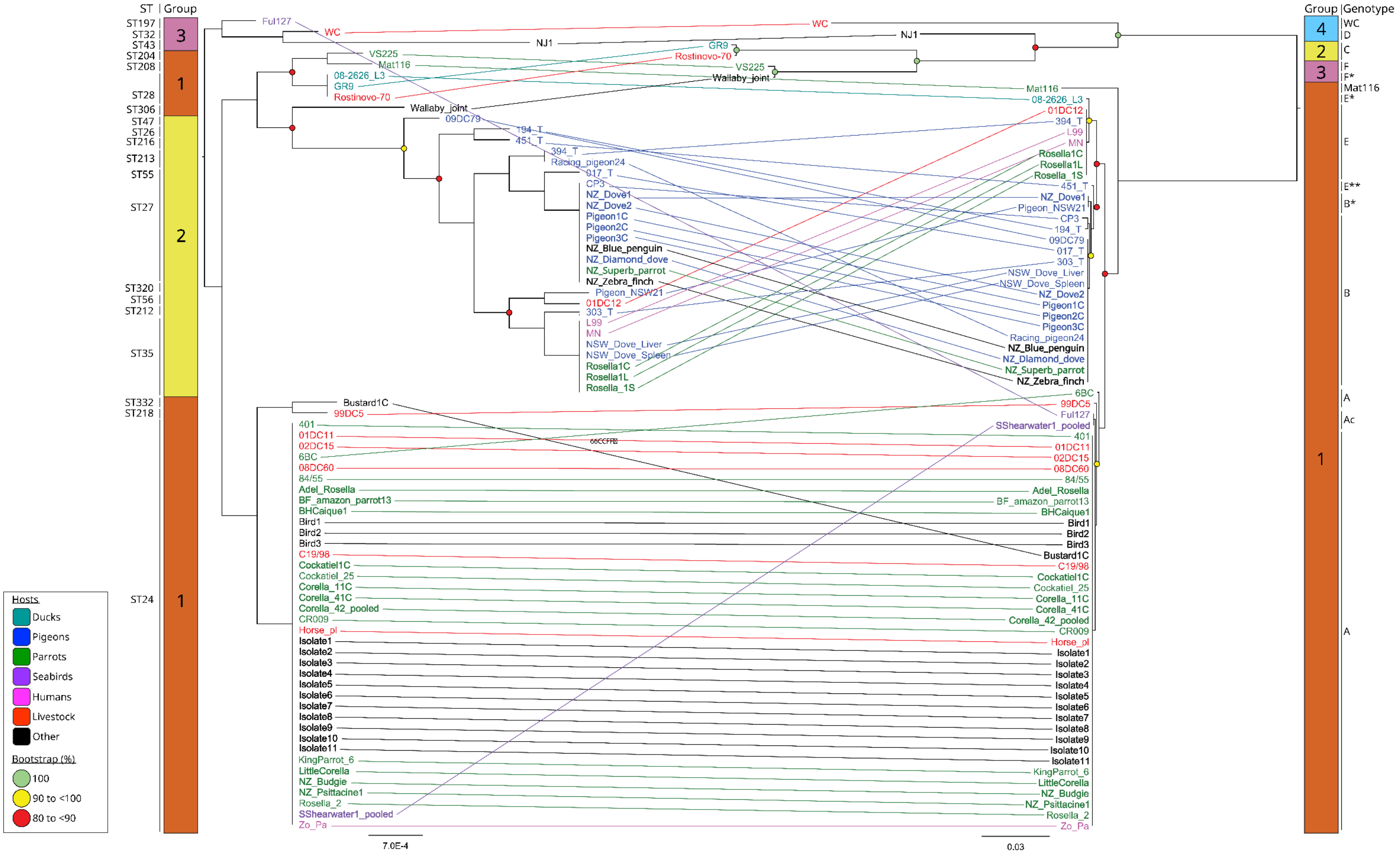

Supplementary Figure S3. Tanglegram comparing MLST and full-length *ompA* gene sequences. A tanglegram comparing a) a 3098 bp alignment representing concatenated MLST sequences, including 36 from this study and 39 reference MLSTs; and (b) a 1085 bp alignment of *ompA* sequences, including 36 from this study and 39 reference sequences. Both phylogenies are midpoint rooted. Branch lengths represent the nucleotide substitutions per site, as indicated by the scale bar. Bootstrap values (using 1000 replicates) are shown. Strains from this study are in bold. Sequences are coloured according to their respective hosts. The outer blocks reflect hierBAPS-defined Phylogenetic Groups (PGs). Major ST is denoted next to the PGs.
